# Nonlinear relationship between body roundness index and prevalence of rheumatoid arthritis in American adults

**DOI:** 10.3389/fnut.2025.1585318

**Published:** 2025-07-18

**Authors:** Xingze Cao, Yongtao Tan, Qiufeng Feng, Pei Ye, Hui Sun, Xuehui Zang

**Affiliations:** ^1^Department of Orthopedics, The Sixth Affiliated Hospital, School of Medicine, South China University of Technology, Foshan, China; ^2^Department of Operation Room, First People's Hospital of Foshan, Foshan, China

**Keywords:** body roundness index, rheumatoid arthritis, adiposity, NHANES, cross-sectional study

## Abstract

**Background:**

The Body Roundness Index (BRI) assesses obesity and fat distribution, yet its correlation with rheumatoid arthritis (RA) is unclear. This study investigated the association between BRI and RA prevalence.

**Methods:**

Using NHANES data from 2011–2018, we conducted a cross-sectional analysis. Logistic regression assessed the BRI-RA relationship, adjusting for various variables. Restricted cubic splines and threshold saturation analysis explored nonlinear associations. Subgroup and sensitivity analyses confirmed the robustness of findings.

**Results:**

A total of 19,875 participants were included in the cross-sectional study. Participants with RA had significantly higher BRI compared with non-RA participants. Logistic regression showed that BRI was positively associated with RA prevalence (OR = 1.14, 95% CI = 1.10–1.17). This positive association remained stable after the inclusion of different covariates (OR = 1.07, 95% CI = 1.02–1.13). Threshold saturation analysis determined a critical BRI value of 5.47, below which the association was strong and above which the association was weakened. Subgroup and sensitivity analyses were consistent with the results of this study.

**Conclusion:**

In American adults, higher BRI levels are significantly associated with RA prevalence. Monitoring BRI may help identify individuals at high risk for RA, providing a new perspective on health management.

## 1 Background

Rheumatoid arthritis is a chronic systemic autoimmune disease with a complex multifactorial pathogenesis, including genetic susceptibility, environmental triggers, and immune system dysregulation, which together contribute to persistent synovial inflammation and structural joint damage (1, 2). RA affects around 0.5 to 1% of the worldwide population, with female being more often affected than males (3, 4). As the population ages, the prevalence of RA is expected to increase, challenging public health systems. RA affects not only the joints but also multiple organs and systems, including the skin, eyes, and heart, significantly impacting patients’ quality of life (5–7). Moreover, RA is associated with an increased risk of systemic comorbidities such as type 2 diabetes and cardiovascular diseases (8, 9). Chronic systemic inflammation in RA promotes insulin resistance, endothelial dysfunction, and atherosclerosis progression, contributing to elevated morbidity and mortality from these conditions (5, 10). Additionally, RA results in long-term medical resource consumption and productivity loss, substantially increasing the socioeconomic burden (11, 12). Given the negative impact of RA on individuals and society, exploring its risk factors and early identification is crucial for preventing and delaying RA progression, reducing patient burden, and improving public health.

Recently, there has been increased emphasis on the relationship between obesity and RA in the field of research. Obesity, a chronic disease that is prevalent and characterized by an excess of body fat, is associated with a variety of health problems, such as cardiovascular illness and type 2 diabetes (13–15). Traditionally, the measurement of obesity has relied on the use of body mass index (BMI) (16). Nevertheless, BMI has inherent limitations since it cannot precisely depict the distribution and ratio of body fat, nor can it distinguish between muscle and fat mass (17). The body roundness index (BRI), a new assessment tool, has gradually attracted increasing attention. Combining body height (BH) and waist circumference (WC), the BRI better reflects body roundness and abdominal fat accumulation (18). Over the past 20 years, BRI among American adults has shown an upward trend (19). The U-shaped relationship between the BRI and all-cause mortality is supported by research demonstrating that the BRI is a noninvasive screening instrument for mortality risk (19, 20). Numerous studies have suggested that the BRI has broad applications in clinical practice (18, 21, 22). Previous research has demonstrated that BRI is a unique risk factor for metabolic syndrome, and there is a nonlinear correlation between BRI levels and the prevalence of metabolic syndrome (23, 24). Similarly, studies have shown that BRI is positively correlated with the prevalence of osteoarthritis, suggesting its potential significance in inflammatory diseases (25, 26). RA is a chronic systemic autoimmune disorder characterized by complex pathophysiological mechanisms closely associated with inflammation (27, 28). Nevertheless, there is a dearth of evidence on the correlation between the BRI and RA. This research utilizes NHANES data from 2011 to 2018 to examine the correlation between the BRI and RA, providing a scientific foundation for future public health initiatives.

## 2 Methods

### 2.1 Survey description

The NHANES is a cross-sectional survey that evaluates the nutritional and health status of the United States demographic. The investigation was approved by the Ethics Review Board, and all participants provided written informed consent. A complex, multistage probability sampling method ensures the representativeness and accuracy of the sample. This study included data from the 2011–2018 NHANES cycles. Subjects younger than 20 years, lacking arthritis data, or lacking BRI data were excluded.

### 2.2 Calculation of BRI

BRI was calculated using height and waist circumference, both measured by trained technicians at Mobile Examination Centers (MECs) following NHANES standardized protocols. Participants removed shoes and outer garments before assessment. BH was measured using a stadiometer, and WC was measured at the midpoint between the lower rib margin and the iliac crest with the participant standing and breathing normally. The BRI was calculated by combining BH and WC using the following formula:


$$\mathrm{BRI}=364.2-365.5\times \sqrt{1-{\left(\frac{\mathrm{WC}(\mathrm{cm})/2\pi }{0.5\times \mathrm{BH}(\mathrm{cm})}\right)}^{2}}$$

### 2.3 Definition of RA

RA was assessed using a validated self-report questionnaire (28, 29). Participants answered, “Has a doctor ever diagnosed you with arthritis?” The question “What type of arthritis?” was posed to those who responded “yes.” All participants who responded “RA” were categorized as RA patients, while the remaining participants were classified as non-RA patients.

### 2.4 Covariates

To control for potential confounding, a range of covariates were included in the adjusted models, encompassing demographic characteristics, lifestyle factors, health conditions, and laboratory biomarkers. Demographic variables included age (<50 or ≥50 years), sex (male or female), race (Mexican American, non-Hispanic White, non-Hispanic Black, and others), poverty income ratio (PIR; <1.0, 1.0–3.0, and >3.0), and education level (under high school, high school, and above high school) (30, 31). Lifestyle variables included smoking status, alcohol consumption, and physical activity. Participants who had smoked ≥100 cigarettes in their lifetime were classified as smokers (32). Drinking status was determined based on whether participants had ever consumed more than 12 alcoholic drinks (33). Physical activity was assessed using the Global Physical Activity Questionnaire and quantified in metabolic equivalent task (MET) minutes per week; participants with <600 MET-min/week were considered physically inactive (34). Health status, including diabetes and chronic kidney disease (CKD), was determined by doctor-diagnosed records or self-reports. Coronary artery disease (CAD) was identified by self-reported coronary artery disease, heart failure, and angina. Laboratory biomarkers were obtained from blood samples collected at Mobile Examination Centers (MECs) and included serum uric acid (SUA), blood urea nitrogen (BUN), alanine aminotransferase (ALT), aspartate aminotransferase (AST), high-density lipoprotein (HDL), total cholesterol (TC), red blood cell (RBC) count, and white blood cell (WBC) count. All measurements followed NHANES quality control protocols and laboratory procedures.

### 2.5 Statistical analysis

This study utilized data from the 2011–2018 cycles of the NHANES, selecting adult participants aged ≥20 years with complete data on BRI and RA status. Descriptive statistics were conducted to compare baseline characteristics between RA and non-RA participants. Weighted logistic regression models were then used to examine the association between the BRI and the prevalence of RA. Weighted multivariable logistic regression models were used to examine the association between BRI and RA prevalence, accounting for the complex sampling design of NHANES. A series of stepwise-adjusted models were constructed to control for potential confounders, including demographic characteristics, lifestyle behaviors, comorbid conditions, and laboratory indicators. To assess potential dose–response patterns, BRI was categorized into quartiles (Q1: <3.93, Q2: 3.93–5.21, Q3: 5.21–6.83, Q4: >6.83) based on its distribution. This classification enabled comparison across exposure levels without assuming a linear relationship. To further explore nonlinearity, restricted cubic spline (RCS) regression models were applied. Threshold saturation analysis was then performed to identify a potential inflection point in the BRI-RA association, followed by two-piecewise logistic regression to estimate the effect on either side of the threshold. Subgroup analyses stratified by age, sex, race/ethnicity, smoking status, and physical activity were conducted to explore potential effect modification. Sensitivity analyses were performed by excluding participants with extreme BRI values (±3 SD) to assess the robustness of the results. All statistical analyses were conducted using R software (version 4.2.3) by employing a significance level of *p* < 0.05.

## 3 Results

### 3.1 Baseline characteristics

**Figure 1:**
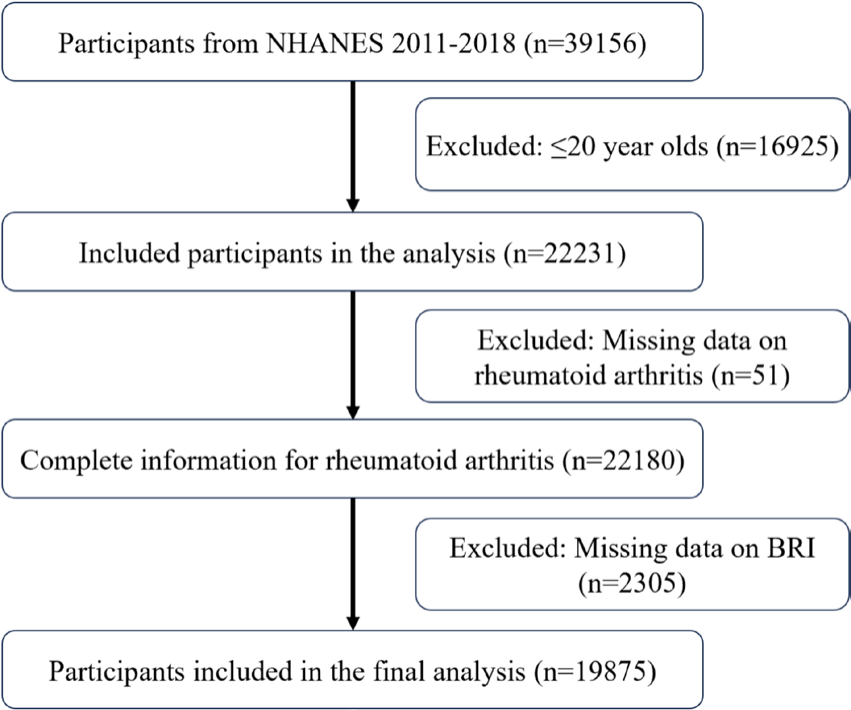
Include participants in the process.

Figure 1 displays the inclusion process with 19,875 participants, including 18,881 non-RA participants and 994 RA participants. Table 1 provides a comprehensive overview of the baseline characteristics. Compared to non-RA participants, RA patients were older, more frequently female, non-Hispanic Black, less educated, and had lower incomes. Their lifestyles included smoking, drinking, and physical inactivity. These results suggest a correlation between RA and an elevated BRI.

**Table 1:** Baseline characteristics of the study population.

Characteristic	Group	Overall	Non-RA	RA	*p*-value
*n*		19,875	18,881	994	
Age (%)	<50	10,240 (51.5)	10,021 (53.1)	219 (22.0)	<0.001
	>50	9,635 (48.5)	8,860 (46.9)	775 (78.0)	
Sex (%)	Female	10,196 (51.3)	9,626 (51.0)	570 (57.3)	<0.001
	Male	9,679 (48.7)	9,255 (49.0)	424 (42.7)	
Race (%)	Mexican American	2,686 (13.5)	2,545 (13.5)	141 (14.2)	<0.001
	Non-Hispanic Black	4,491 (22.6)	4,187 (22.2)	304 (30.6)	
	Non-Hispanic White	7,332 (36.9)	6,986 (37.0)	346 (34.8)	
	Others	5,366 (27.0)	5,163 (27.3)	203 (20.4)	
Education level (%)	Under high school	4,324 (21.8)	4,028 (21.3)	296 (29.8)	<0.001
	High school	4,381 (22.0)	4,145 (22.0)	236 (23.7)	
	Above high school	11,157 (56.1)	10,697 (56.7)	460 (46.3)	
	No record	13 (0.1)	11 (0.1)	2 (0.2)	
PIR (%)	<1	3,796 (21.3)	3,526 (20.8)	270 (30.3)	<0.001
	1–3	7,511 (42.1)	7,119 (42.0)	392 (44.0)	
	>3	6,554 (36.7)	6,325 (37.3)	229 (25.7)	
Smoke (%)	No	11,332 (57.0)	10,863 (57.5)	469 (47.2)	<0.001
	Yes	8,530 (42.9)	8,007 (42.4)	523 (52.6)	
	No record	13 (0.1)	11 (0.1)	2 (0.2)	
Drinking (%)	No	4,382 (23.8)	4,126 (23.6)	256 (27.1)	0.040
	Yes	14,054 (76.2)	13,366 (76.4)	688 (72.8)	
	No record	12 (0.1)	11 (0.1)	1 (0.1)	
Activity status (%)	Active	10,488 (52.8)	10,042 (53.2)	446 (44.9)	<0.001
	Inactive	9,387 (47.2)	8,839 (46.8)	548 (55.1)	
Diabetes (%)	No	16,606 (83.6)	15,920 (84.3)	686 (69.0)	<0.001
	Yes	2,729 (13.7)	2,464 (13.1)	265 (26.7)	
	No record	540 (2.7)	497 (2.6)	43 (4.3)	
CKD (%)	No	19,169 (96.4)	18,257 (96.7)	912 (91.8)	<0.001
	Yes	684 (3.4)	604 (3.2)	80 (8.0)	
	No record	22 (0.1)	20 (0.1)	2 (0.2)	
CAD (%)	No	18,524 (93.2)	17,689 (93.7)	835 (84.0)	<0.001
	Yes	1,351 (6.8)	1,192 (6.3)	159 (16.0)	
SUA [mean (SD)] (mg/dL)		5.43 (1.44)	5.42 (1.43)	5.63 (1.66)	<0.001
HDL [mean (SD)] (mmol/L)		1.38 (0.42)	1.38 (0.42)	1.39 (0.42)	0.475
TC [mean (SD)] (mmol/L)		4.93 (1.08)	4.94 (1.08)	4.91 (1.08)	0.454
BUN [mean (SD)] (mmol/L)		5.00 (2.12)	4.97 (2.09)	5.53 (2.59)	<0.001
ALT [mean (SD)] (U/L)		24.58 (20.54)	24.64 (20.78)	23.50 (15.35)	0.096
AST [mean (SD)] (U/L)		24.85 (16.87)	24.83 (16.91)	25.11 (16.12)	0.626
WBC [mean (SD)] (1,000 cells/uL)		7.26 (3.76)	7.26 (3.82)	7.34 (2.56)	0.489
RBC [mean (SD)] (million cells/uL)		4.67 (0.50)	4.68 (0.50)	4.55 (0.51)	<0.001
WC [mean (SD)] (cm)		99.81 (16.57)	99.53 (16.53)	105.10 (16.41)	<0.001
BH [mean (SD)] (cm)		166.77 (10.12)	166.87 (10.11)	164.87 (10.12)	<0.001
BMI [mean (SD)] (kg/m^2^)		29.28 (6.95)	29.19 (6.92)	31.02 (7.29)	<0.001
BRI [mean (SD)]		5.62 (2.40)	5.57 (2.38)	6.52 (2.52)	<0.001

Mean (SD) for continuous variables, % for categorical variables. ALT, Alanine Aminotransferase; AST, Aspartate Aminotransferase; BMI, Body Mass Index; BH, Body height; BRI, Body Roundness Index; BUN, Blood Urea Nitrogen; CAD, Coronary Artery Disease; CKD, Chronic Kidney Disease; RA, Rheumatoid arthritis; RBC, Red blood cell; SUA, Serum uric acid; TC, Total Cholesterol; TG, Triglyceride; WBC, White blood cell, WC, Waist Circumference.

### 3.2 Association between BRI and prevalence of RA

Logistic regression analysis illustrates the correlation between the BRI and the prevalence of RA in Table 2. The prevalence of RA was significantly associated with BRI (OR = 1.14, 95% CI = 1.10–1.17), as indicated by the univariate model. A significant association between BRI and the prevalence of RA persisted after accounting for covariates. The prevalence of RA increased by 7% for each unit increase in BRI, as indicated by Model 3 (OR = 1.07, 95% CI = 1.02–1.13). Additionally, the prevalence of RA was substantially higher in the highest BRI quartile than in the lowest quartile (OR = 1.91, 95% CI = 1.22–2.97). The robustness of the association between the BRI and prevalence of RA is suggested by these findings.

**Table 2:** The relationship between BRI and RA.

		Model 1 OR (95%CI) *p*-value	Model 2 OR (95%CI) *p*-value	Model 3 OR (95%CI) *p*-value
RA	BRI	1.14 (1.10, 1.17) < 0.001	1.10 (1.06, 1.15) < 0.001	1.07 (1.02, 1.13) 0.009
	Q1	[Reference]	[Reference]	[Reference]
	Q2	1.98 (1.32, 2.98) < 0.001	1.64 (1.09, 2.45) 0.018	1.61 (1.03, 2.52) 0.037
	Q3	2.67 (1.85, 3.87) < 0.001	2.02 (1.39, 2.93) < 0.001	1.86 (1.29, 2.68) 0.002
	Q4	3.15 (2.23, 4.45) < 0.001	2.29 (1.58, 3.31) < 0.001	1.91 (1.22, 2.97) 0.006
	P for trend	<0.001	<0.001	0.013

BRI, Body Roundness Index; CI, Confidence Interval; OR, Odds Ratio; RA, Rheumatoid arthritis. Model 1, No covariates adjusted; Model 2, Adjusted for Age, Sex, and Race; Model 3, Adjusted for age, Sex, Race, PIR, Educational level, Smoke, Drinking, Activity status, CAD, CKD, Diabetes, SUA, BUN, ALT, AST, HDL, TC, WBC, RBC.

### 3.3 Nonlinear relationship between BRI and RA

**Figure 2:**
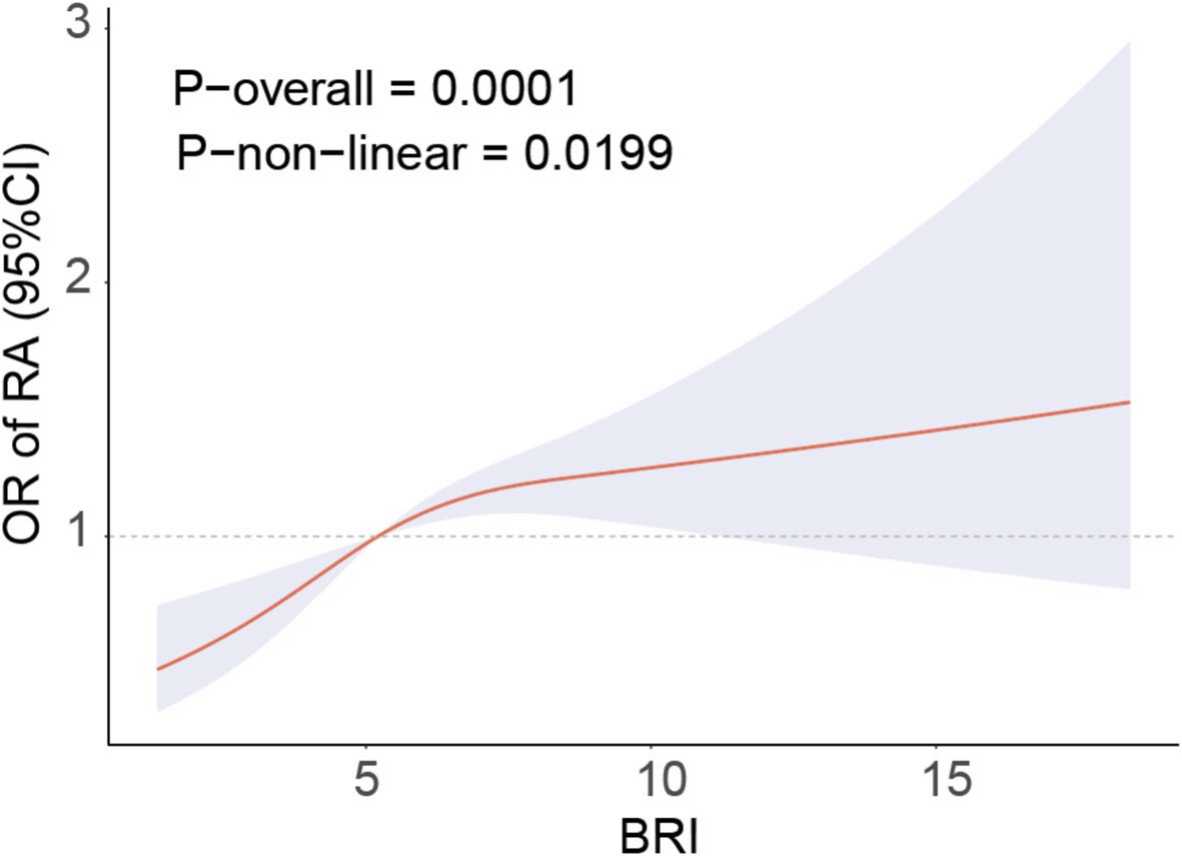
RCS curve fits the Association of BRI with RA. Adjusted for age, Sex, Race, BMI, Educational level, Smoke, Drinking, Activity status, CAD, CKD, Diabetes, Calcium, Phosphorus, SUA, BUN, ALT, AST, HDL, TC.

Figure 2 demonstrates the non-linear correlation between BRI and RA, indicating that a greater BRI is strongly linked to a higher occurrence of RA. Threshold saturation analysis identified a critical point at BRI = 5.47, with Table 3 presenting the two-piece logistic regression based on this point. On the left side of the critical point (BRI < 5.47), each unit increase in BRI increased the prevalence of RA by 30%. The prevalence of RA was not significantly affected by variations in the BRI on the right side of the critical point. These results indicate a substantial correlation between the BRI and the prevalence of RA within a particular range.

**Table 3:** Analysis of the BRI saturation effect and RA.

	BRI (%)	OR (95%CI) *p*-value
RA	Standard linear model	1.09 (1.05, 1.12) < 0.001
	BRI < 5.47	1.30 (1.17, 1.45) < 0.001
	BRI > 5.47	1.04 (1.00, 1.08) 0.065
	Log-likelihood ratio test	<0.001

BRI, Body Roundness Index; CI, Confidence Interval; OR, Odds Ratio; RA, Rheumatoid arthritis. Adjusted for age, Sex, Race, PIR, Educational level, Smoke, Drinking, Activity status, CAD, CKD, Diabetes, SUA, BUN, ALT, AST, HDL, TC, WBC, RBC.

### 3.4 Subgroup and sensitivity analyses

**Figure 3:**
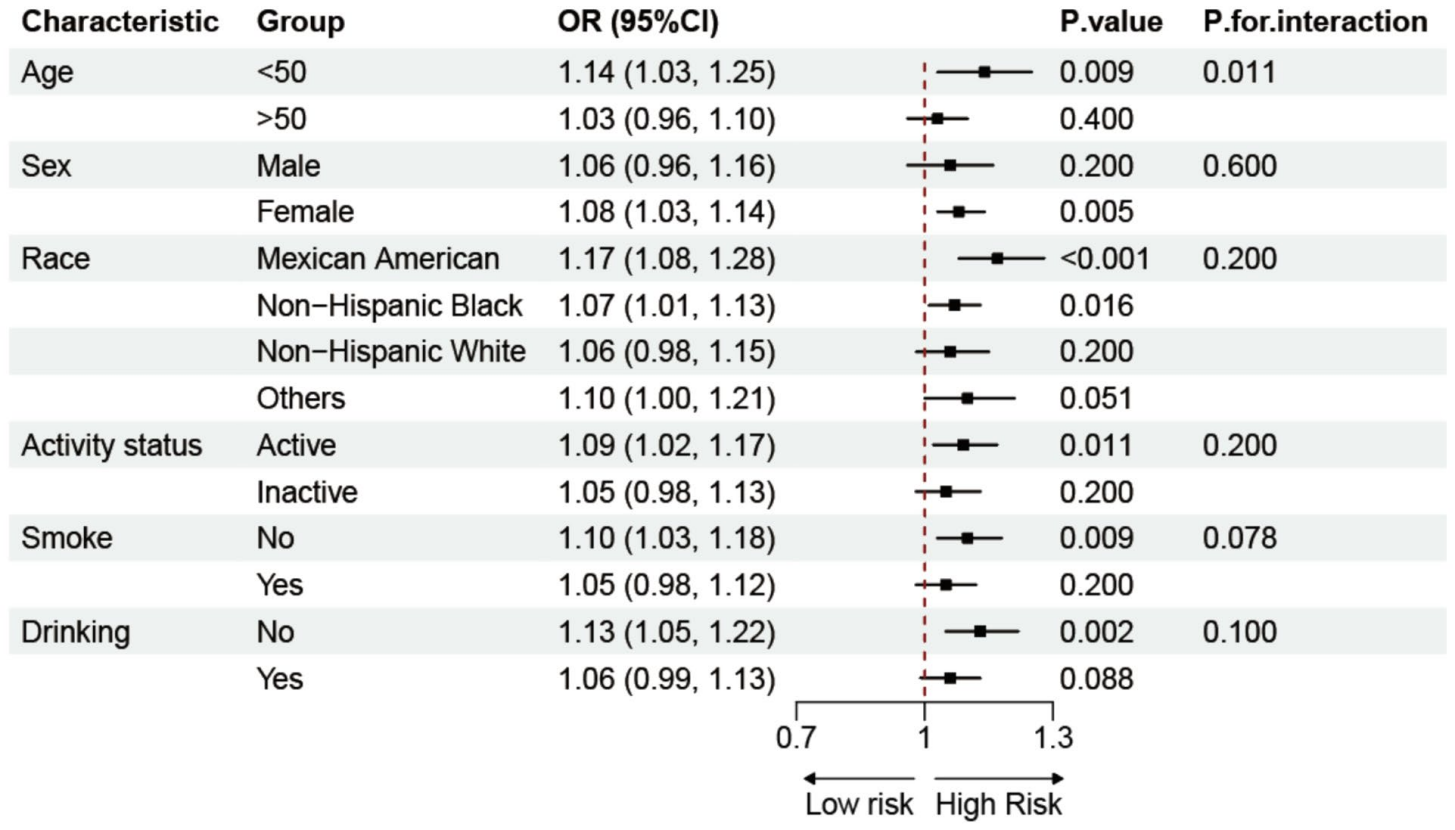
Subgroup analysis of the association between BRI and RA.

Subgroup analyses based on demographics and lifestyle factors explored the potential relationship between BRI and RA, as shown in Figure 3. The results demonstrated a constant positive correlation between BRI and prevalence of RA in various groups, hence enhancing the study’s credibility. Interaction tests revealed a stronger association between the BRI and prevalence of RA in populations younger than 50 years. After excluding extreme BRI ± 3SD values, a sensitivity analysis was conducted on the remaining 19,621 participants. The data shown in Supplementary Table 1 validate the positive correlation between the BRI and prevalence of RA, which aligns with the study’s conclusions.

## 4 Discussion

This study revealed a significant association between the BRI and prevalence of RA among American adults, indicating a strong correlation within a specific range. Subgroup and sensitivity analyses confirmed this finding. These findings have important clinical implications. Regular monitoring of BRI can help identify high-risk groups and provide new directions for early intervention to slow the progression of RA.

Recently, the association between obesity and RA has garnered widespread attention. Obesity is a major public health crisis due to its prevalence and associated adverse health outcomes (13). Mendelian studies suggest that BMI, a traditional measure of obesity, has a causal relationship with increased RA risk (35). However, multiple studies have indicated that BMI assessment of obesity may lead to the obesity paradox (36, 37). WC, an indicator of abdominal obesity, is correlated with increased RA risk in women (38). However, considering WC alone without height measurements may lack persuasiveness. The BRI may be a better composite measure for assessing disease. While BMI is widely used to assess general obesity, it fails to differentiate between fat and lean mass or to capture fat distribution patterns that are critical for understanding inflammatory risk (39). Abdominal and visceral adiposity are particularly important because they function as metabolically active endocrine organs, secreting proinflammatory adipokines, which maintain a chronic low-grade inflammatory state—a process often described as lipidemic stress (40, 41). This persistent inflammation may contribute to immune dysregulation and increase susceptibility to autoimmune diseases such as RA. In contrast, BRI integrates waist circumference and height, providing a more precise assessment of central adiposity and its metabolic consequences. Studies have shown that the BRI more accurately reflects body fat and predicts disease than traditional indices such as BMI, WC, and BH (18, 42). Our findings are consistent with previous literature, showing a strong association between BRI and prevalence of RA within a specific range, with diminished effects beyond the threshold. High BRI levels are often associated with diseases such as diabetes and cardiovascular diseases (21, 43, 44), which may increase the prevalence of RA and accelerate its progression, weakening the effect of the BRI on the prevalence of RA. Subgroup analysis revealed an age interaction effect on the BRI-RA relationship, with BRI showing no significant effect in older populations. This may be because older individuals typically have more chronic diseases, masking the independent effect of the BRI on prevalence of RA (45, 46). This finding provides insights into early RA prevention and intervention and offers a comprehensive assessment method for managing obesity-related diseases. In addition, our study observed that the positive association between BRI and RA prevalence was more pronounced among women. This finding aligns with existing epidemiological evidence indicating that RA risk is consistently higher in females. Such sex differences may be explained by several biological mechanisms (4). Estrogen can influence immune regulation by enhancing B cell activation and promoting autoantibody production, thereby increasing the level of autoimmune responses (47). Females also tend to exhibit higher overall immune reactivity, which may reduce tolerance to self-antigens and increase the risk of autoimmune diseases (48). Moreover, the X chromosome contains numerous immune-related genes that may have greater pathogenic potential in females (49). These mechanistic features help to interpret the higher prevalence of RA observed in women and underscore the importance of considering individual characteristics such as age and sex in RA risk assessment, early identification, and prevention strategies to support more targeted health management.

Beyond these epidemiological associations, the underlying pathophysiological mechanisms can be explained from several perspectives. The evidence indicates that obesity is a critical factor in the pathophysiology of RA (50, 51). Research suggests that obesity contributes to the inflammation of adipocytes in white adipose tissue (WAT), which in turn decreases the production of anti-inflammatory factors and increases the secretion of proinflammatory factors such as TNF-*α*, IFN-*γ*, IL-1β, and IL-6 (52). These proinflammatory factors promote T-cell differentiation and activation, which may increase RA risk by stimulating T-cell immune activity (53, 54). The excessive accumulation of reactive oxygen species (ROS) in cells, which results in the impairment of DNA and protein function, is a consequence of obesity-induced oxidative stress (55–57). Oxidative stress triggers a strong immune response, releasing inflammatory mediators such as NF-κB, activating the NLRP3 inflammasome, and controlling T-cell differentiation, exacerbating RA symptoms and severity (58, 59). Additionally, obesity is often accompanied by hyperglycemia and hyperinsulinemia, leading to insulin resistance and inflammatory responses and aggravating RA progression (60). Although balanced insulin has anti-inflammatory effects, RA itself can induce insulin resistance, creating a bidirectional interaction (61). In summary, the BRI-RA relationship may result from multifactorial interactions. Additional research is required to investigate the pathophysiological mechanisms involved.

There are numerous advantages to this study. First, the large sample size and rigorous random sampling ensured that the findings represented the entire U. S. population. Second, various covariates were included in stepwise adjustment models, enhancing the reliability of the results. Additionally, a threshold effect between the BRI and prevalence of RA was identified, showing a strong association within a specific range. Nevertheless, this study has certain limitations. First, the cross-sectional design does not allow for the determination of a causal relationship between BRI and RA. Second, the diagnosis of RA relied on self-reported data from participants, which may be subject to recall bias. Additionally, as this study was conducted in a U. S. population, its applicability to other ethnic groups remains unverified. Therefore, large-scale longitudinal studies are needed in the future to further validate these findings and assess their generalizability across diverse populations.

## 5 Conclusion

In American adults, higher BRI levels are significantly associated with RA prevalence. Monitoring BRI may help identify individuals at high risk for RA, providing a new perspective on health management.

## Data Availability

Publicly available datasets were analyzed in this study. This data can be found here: https://wwwn.cdc.gov/nchs/nhanes/default.aspx.
