# Supplementary material for: Nonlinear relationship between body roundness index and prevalence of rheumatoid arthritis in American adults
Source: Front Nutr. 2025 Jul 18;12:1585318. doi: 10.3389/fnut.2025.1585318 (PMC12313493; doi:10.3389/fnut.2025.1585318)
Supplement: Supplementary file 1 [file Table_1.DOCX]

**Supplementary Table 1 Sensitivity analysis between BRI and RA.**

|  |  | Model 1  OR (95%CI) P-value | Model 2  OR (95%CI) P-value | Model 3  OR (95%CI) P-value |
| --- | --- | --- | --- | --- |
| RA | BRI | 1.16 (1.12, 1.20) <0.001 | 1.12 (1.07, 1.17) <0.001 | 1.09 (1.02, 1.15) 0.009 |
|  | Q1 | [Reference] | [Reference] | [Reference] |
|  | Q2 | 1.98 (1.32, 2.98) <0.001 | 1.64 (1.09, 2.46) 0.018 | 1.62 (1.04, 2.53) 0.036 |
|  | Q3 | 2.67 (1.85, 3.87) <0.001 | 2.03 (1.40, 2.94) <0.001 | 1.86 (1.29, 2.68) 0.002 |
|  | Q4 | 3.10 (2.19, 4.37) <0.001 | 2.25 (1.55, 3.27) <0.001 | 1.90 (1.22, 2.98) 0.006 |
|  | P for trend | <0.001 | <0.001 | 0.014 |

CI: Confidence Interval; OR: Odds Ratio; Q: Quartiles; BRI: Body Roundness Index

Model 1: No covariates adjusted; Model 2: Adjusted for Age, Sex, and Race; Model 3: Adjusted for age, Sex, Race, PIR, Educational level, Smoke, Drinking, Activity status, CAD, CKD, Diabetes, SUA, BUN, ALT, AST, HDL, TC, WBC, RBC.
